# Intermittent dosing of zoledronic acid based on bone turnover marker assessment reduces vertebral and non-vertebral fractures

**DOI:** 10.1093/jbmrpl/ziae072

**Published:** 2024-05-31

**Authors:** Tove Tveitan Borgen, Sindre Lee-Ødegård, Barbara Fink Eriksen, Erik Fink Eriksen

**Affiliations:** Department of Rheumatology, Drammen Hospital, N-3004 Drammen, Norway; Department of Endocrinology, Morbid Obesity and Preventive Medicine, Oslo University Hospital, 0424 Oslo, Norway; Faculty of Medicine, University of Aarhus, 8000 Aarhus, Denmark; Spesialistsenteret Pilestredet Park, 0176 Oslo, Norway; Spesialistsenteret Pilestredet Park, 0176 Oslo, Norway; Faculty of Dentistry, University of Oslo, 1142 Blindern, Oslo, Norway

**Keywords:** antiresorptives, biochemical markers of bone turnover

## Abstract

Previous studies have demonstrated that the administration of zoledronic acid (ZOL) once yearly for 3 years or once over 3 years, yields similar antifracture efficacy. Bone turnover markers can predict the antifracture efficacy of antiresorptive agents, with procollagen type 1 N-terminal propeptide (P1NP) being the most useful marker. In this retrospective cohort study, we explored the effects of intravenous dosing of ZOL guided by serum (S)-P1NP assessment on bone mineral density (BMD) and fractures. Consenting patients (*N* = 202, mean age 68.2 years) with osteoporosis were treated with ZOL for an average of 4.4 (range 2-8) years. S-P1NP and BMD were measured at baseline and every 1-2 years. We assessed the number of subsequent vertebral and nonvertebral fractures in the 2-year time periods. The number of patients assessed was 202, 147, 69, and 29 at years 1-2, 3-4, 5-6, and 7-8, respectively. A new ZOL infusion was given if S-P1NP exhibited values above 35 μg/L. BMD increased by 6.2% (SD 4.0) over the first 2 years and stabilized in years 2-8 (*P* <.05). Median S-P1NP exhibited an initial reduction from 58.0 to 31.3 μg/L at year 2 and then increased to 39.0 μg/L at years 7-8. Compared with fractures observed in the last 2 years before baseline, fracture rates exhibited consistent reductions, for vertebral fractures odds ratio (OR) [95% confidence interval] = 0.61 [0.47, 0.80], *P* <.001 and for nonvertebral fractures OR = 0.23 [0.18, 0.31], *P* <.001. In conclusion, intermittent dosing of intravenous ZOL based on the assessment of S-P1NP with cut-off at 35 μg/L resulted in an initial increase followed by a stable BMD, suppression of S-P1NP, and stable reduction of fractures for 8 years. Only 39% of patients needed more than one infusion. This approach reduces healthcare costs and might also reduce the risk of rare side effects such as osteonecrosis of the jaw and atypical femoral fracture.

## Introduction

Newer studies have demonstrated that levels of bone turnover markers (BTMs) reflect the antifracture efficacy of antiresorptive drugs (ARDs).^1-3^ Procollagen type 1 N-terminal propeptide (P1NP) appears to be the clinically most useful marker,^1^ as it is temperature stable, shows no significant diurnal variation in treated patients, has good ability to discriminate patients adherent to ARD or not, and can predict bone mineral density (BMD) increases and fractures in patients on ARD.^4^

Bisphosphonates exhibit prolonged action, which is exploited by using drug holidays during treatment.^5^ Furthermore, substudies from the Health Outcomes and Reduced Incidence with Zoledronic Acid Once Yearly (HORIZON) trial, investigating clinical effects of i.v. zoledronic acid (ZOL) and other later studies, have demonstrated that dosing intervals exceeding 1 year may exert significant antifracture efficacy.^6-9^ In the HORIZON trial, exploring the antifracture efficacy of yearly infusions of ZOL, 1.367 patients only received one infusion.^6^ Follow-up of these patients revealed similar reductions in vertebral and nonvertebral fracture incidence than seen in the majority, who received yearly infusions for 3 years. Over 3 years, clinical fractures were reduced by 32% in the single infusion groups vs. 34% in the once yearly infusion group. Morphometric vertebral fractures were reduced by 68% and 70%, respectively.^6^ Additionally, more infrequent dosing of ZOL may reduce the risk of rare, but severe adverse effects such as osteonecrosis of the jaw and atypical femoral fractures.^10^^,^^11^ In substudies from the HORIZON-Pivotal Fracture Trial (HORIZON-PFT), it emerged that BTMs were more sensitive markers of bisphosphonate action, as 1-year changes in BTM yielded a larger percentage of treatment effect than changes in BMD after 3 years.^12^ Despite this knowledge, research on how to use BTMs to time the frequency of ZOL infusions is scarce, albeit less frequent dosing of ZOL can have many advantages. Our clinical experience has shown that the duration of BTM suppression after a ZOL infusion varies between patients, and therefore, individually tailored treatment can be required. This prompted us to test, whether intermittent dosing of ZOL based on BTM levels could maintain sufficient antifracture efficacy. Based on the data published by Eastell *et al.*,^2^^,^^13^ we chose a cut-off level for S- P1NP of 35 μg/L to initiate a new infusion.

The detailed objectives were to explore:

1. The effects of iv. dosing of ZOL given at S-P1NP > 35 μg/L on increases in BMD.
2. The effects of iv. dosing of ZOL given at S-P1NP > 35 μg/L on the incidence of vertebral and nonvertebral fractures.
3. The rate of reinfusion of ZOL given at S-P1NP > 35 μg/L.

## Materials and methods

The patients were recruited from Pilestredet Park Osteoporosis Clinic, where they had osteoporosis diagnosed, treated, and followed up. Inclusion criteria were: BMD T-score ≤ −2.5 at the lumbar spine or total hip, with or without low-energy fracture, or BMD T-score < −1.0 with low-energy fracture. Exclusion criteria were: treatment with other anti-osteoporotic agents except hormone replacement therapy (HRT) within the last 5 years, hypocalcemia, or eGFR <35 ml/min. There was no age-limit for inclusion.

Patients had calcium supplementation if they had a daily dietary calcium intake of less than 1000 mg. S-vitamin D was measured at every follow-up and a vitamin D supplementation was prescribed if low levels, to ensure S-vitamin D levels >75 nmol/L (reference level > 50 nmol/L).

We conducted a retrospective cohort study based on medical records from patients who were followed at Pilestredet Park Osteoporosis Clinic, Oslo between 2014 and 2022. Baseline was defined as the time of their first historical ZOL infusion. S-P1NP had been measured before the first dose of ZOL and every 1-2 years, using Elecsys Total P1NP immunoassay on Cobas e411 analyzer (Roche Diagnostics GmbH, Mannheim, Germany) with an intra-assay coefficient of variances (CVs) of 5.0%–5.4% and interassay CVs of 2.0%–4.4%.

BMD was measured using dual X-ray absorptiometry (DXA) of the lumbar spine (L1-L4) and total hip at baseline and at follow-up using iDXA (GE Lunar, Pro, Madison, WI, USA). As this was a study conducted in a clinical setting, the time points of follow-up varied and therefore were divided into slots of years 1-2, 3-4, 5-6, and 7-8.

Fractures were registered for 2 years before baseline and during follow-up based on patient information at every visit. Non-vertebral fractures were defined as clinical fractures at any site, except fractures of the spine, fingers, toes, face, and skull. A lateral scan of the thoracolumbar spine was obtained at every visit using DXA, and vertebral fracture assessment (VFA) performed according to the method of Genant,^14^ and vertebral fractures were reported as yes or no. The fracture incidence during follow-up was compared to the fracture incidence the 2 years before baseline.

The criteria for a new ZOL infusion were S-P1NP levels exceeding 35 μg/L, or decreases in BMD >2% at the hip or spine from the last measurement. The cut-off at S-P1NP 35 μg/L was chosen, as levels in the lower half of the reference interval correspond to BTM response of more than least significant change, and reflect the treatment effect of ARD.^15^ Correspondingly, S-P1NP levels above 35 μg/L were considered as waning of the ZOL effect, indicating time for a new infusion. If patients suffered two or more low-energy fractures during treatment, they were offered to change treatment to either teriparatide or denosumab and were excluded from the study population.

A total of 16 patients exhibited reductions in BMD >2% in total hip or lumbar spine BMD. Nine patients experienced both significant reductions in BMD and two or more fractures and one patient experienced 2 or more fractures only. The majority (23 patients) changed to treatment with denosumab after a mean treatment period of 6.7 years (range 1-12) and a mean of 2.6 ZOL infusions (range 1-5). Two patients changed to teriparatide, and one patient changed to ZOL 4 mg every 6 months, due to a breast cancer diagnosis. The choice between denosumab and teriparatide was based on whether the patients fulfilled reimbursement criteria for osteoanabolic treatment or not.

All patients were included after oral and written consent. The study was approved by the Regional Ethical Committee of the Southeast Region of Norway (ID 218347).

## Statistical analyses

The results were presented as mean ± standard deviation (SD) for the continuous variables and number (%) for categorical variables. We inspected histograms to check the continuous variables for normality. As the distribution of S-P1NP was right skewed, we reported these variables as medians with interquartile range (IQ) and log-transformed when they were used as continuous variables. Log transformation was not employed in the analyses where cut-off values were tested. We used multiple linear regression analyses to investigate differences in continuous variables from baseline to follow-up with adjustments for age and sex.

Fracture rates were summarized by 2-year categories. To obtain a measure of variability given a fixed number of patient years, we assumed that the number of fractures followed a Poisson distribution from which we calculated 95% confidence intervals (95% CI) for the fracture rates. To test the null hypothesis of no change in fracture rates after treatment with ZOL vs. before treatment, we used generalized linear models assuming a Poisson error structure, correcting for over-dispersion when necessary, and included follow-up length as an offset to model rates. We estimated the main effect across all years and also performed pairwise tests for each time point vs. baseline using the *emmeans* R package. Models were developed separately for each type of fracture. Furthermore, sensitivity analyses were performed with adjustment for age, sex, and total hip BMD to explore if these influenced on rates of vertebral and nonvertebral fractures. Statistical analyses were performed using Stata v18 (Version 18, StataCorp LP, TX, USA) and R v4.1.2 (R Core Team, Vienna, Austria).

## Results

Two hundred and two patients were enrolled (89% women) with a mean age of 68.2 (SD 9.9) years (range 35-89 years). Vitamin D, PTH, calcium, and eGFR were within the reference interval. A total of five patients were using low-dose HRT (Estradiol dose of 1 mg/day) during the study period. Follow-up duration varied from 2 to 8 years, and 26 patients (12.7%) discontinued treatment during the observation period. Of the total, 21.5% of the patients exhibited previous vertebral fractures, and 46.5% had previous nonvertebral fractures (Table 1). Baseline demographics revealed S-P1NP levels in the upper premenopausal reference range and the BMD T-scores were −2.4 at the spine and −1.7 at the total hip (Table 2). Within the 2 years before inclusion, 12.4% of the patients had suffered one or more vertebral fractures and 21.8% had suffered one or more nonvertebral fractures.

**Table 1:** Baseline patient characteristics.

Cases, *n*	202
Observation time, years (range)	4.4 (2-8)
Secondary osteoporosis, *n* (%)	32 (16.0)
S-25 hydroxy vitamin D, nmol/L (SD) (ref > 50)	83.3 (24.1)
S-PTH, pmol/L (SD) (ref 2–11)	4.44 (2.38)
S-Calcium, mmol/L (SD) (ref 2.15-2.51)	2.34 (0.12)
S-eGFR, mL/min/1,73m^2^ (SD) (ref > 70)	86.3 (14.7)
History of vertebral fracture any time, *n* (%)	44 (21.5)
History of non-vertebral fracture any time, *n* (%)	94 (46.5)

Abbreviation: Ref, reference interval.

**Table 2:** BMD, serum procollagen type 1 (S-P1NP) and fractures at baseline and follow-up.

	**Baseline**	**Years 1-2**	**Years 3-4**	**Years 5-6**	**Years 7-8**
Individuals observed, *n* Women, *n* (%) Age, years (SD) ZOL infusions, *n* (%)	202 180 (89.1) 68.2 (9.9) 202 (100)	202 171 (91.4) 70.2 (9.7) 62 (30)	147 103 (88.8) 72.6 (8.8) 28 (19)	69 47 (92.1) 75 (9.3) 18 (26)	29 17 (94.4) 80.3 (7.7) 12 (41)
**BMD, observations, *n*** L1-L4, g/cm^2^ (SD) T-score L1-L4 (SD) Increase from baseline, % (SD) Total hip, g/cm^2^ (SD) T-score total hip (SD) Increase from baseline, % (SD)	202 0.901 (0.113) −2,4 (0.9) 0 0.810 (0.093) −1.7 (0.7) 0	187 0.957^c^ (0.113) 6.2 (4.0) 0.829^c^ (0.097) 2.6 (2.3)	116 0.941^c^ (0.110) 7.0 (4.6) 0.820^c^ (0.094) 2.5 (2.7)	51 0.930^c^ (0.077) 6.3 (5.5) 0.809^c^ (0.077) 2.0 (3.7)	18 0.900^c^ (0.085) 8.0 (6.1) 0.801^c^ (0.077) 1.5 (4.3)
**S-P1NP, observations, *n*** S-PINP, μg/L (IQ) S-PINP decrease from baseline, %	191 58.0 (42-70) –	148 31.3^b^ (22-39) 46.0	134 36.5^b^ (29-41) 37.1	64 39.4^b^ (31-48) 32.1	24 39.0 (31.5-46) 32.8
**Incident fractures** Patients with VFX, *n* (%) VFX, *n* VFX reduction from year -2-0 (%) Patients with NVFX, *n* (%) NVFX, *n* NVFX reduction from year -2-0 (%)	**Year -2-0** 25 (12.4) 43 – 44 (21.8) 67 –	17 (8.4) 22 32.3 11 (5.4) 18 75.2	10 (6.8) 11 45.1 6 (4.1) 8 82.2	4 (5.8) 8 53.2 4 (5.8) 4 73.4	1 (3.4) 2 72.6 2 (6.9) 2 68.3

Values are mean ± SD, number (%), and median with IQ. S-PINP, serum procollagen type I. Baseline incident fractures are fractures the last 2 years before baseline. ^a^*P* < .05, ^b^*P* < .01, ^c^*P* < .001, adjusted for sex and age.

### **Treatment with** ZOL

**Figure 1:**
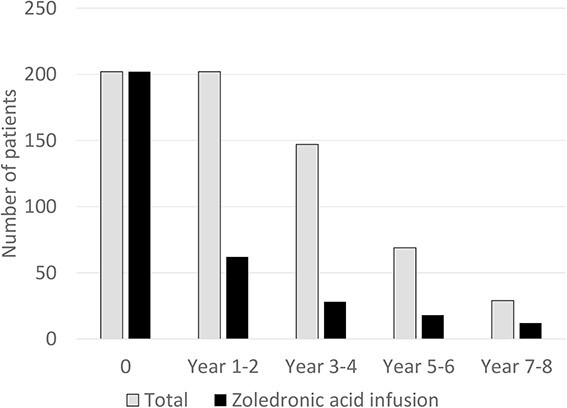
Number of patients followed-up (gray) and number of patients receiving ZOL infusions (black).

All the patients had an intravenous infusion of 5-mg ZOL at baseline. The next infusion was given when S-P1NP values exceeded 35 μg/L, hence 62 (33.2%), 28 (24.1%), 18 (35.3%), and 12 (66.7%) had a new infusion at years 1-2, 3-4, 5-6, and 7-8, respectively (Fig. 1). Mean time from first ZOL infusion at baseline to second ZOL infusion was 2.3 years. The mean observation time was 4.4 years (range 2-8 years). Of the 202 patients, 123 (60.9%) patients only had the baseline infusion and no need for further infusions, 52 (25.7%) had 2 infusions, 13 (6.4%) had 3 infusions, 13 (6.4%) had 4 infusions, and 1 patient had 5 infusions (Table 3). Of the 78 patients who got new infusions, 30, 19, 12, 10, 5, 1, and 1 got the second infusion after 1, 2, 3, 4, 5, 6, and 7 years, respectively (Table 3). Twelve patients had three annual infusions from baseline.

**Table 3:** Total number of ZOL infusions per patient and time from first to second infusion.

Total ZOL infusions, *n*	1	2	3	4	5	6	7
Patients, *n*	123	52	13	13	1	0	0
**Time from first to second infusion, year**	**1**	**2**	**3**	**4**	**5**	**6**	**7**
Patients, *n*	30	19	12	10	5	1	1

### BMD and S-P1NP

Lumbar spine BMD increased by 6.2 (SD 4.0)% (*P* < .001) during the first 2 years and remained elevated until years 7-8, when only 29 patients were assessed (*P* < .001) (Table 2). Similarly, total hip BMD exhibited an increase of 2.6 (SD 2.3)% during the first 2 years and remained elevated until years 5-8.

The bone turnover marker S-P1NP remained suppressed during the whole treatment period with reductions ranging between 46% and 32% (Table 2).

### Vertebral and nonvertebral fractures

**Figure 2:**
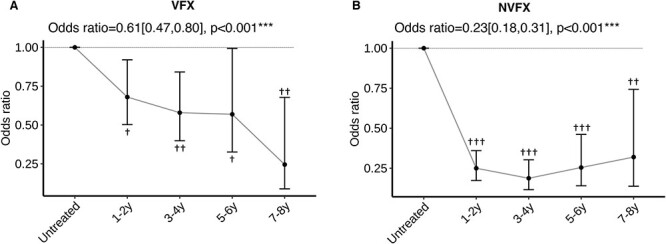
Effects of ZOL on (A) vertebral fracture rates and (B) nonvertebral fracture rates. ^†^*P* < .05, ^††^*P* < .01, and ^†††^*P* < .001 vs. untreated. Error bars are 95% confidence intervals. Abbreviation: Y, year.

Compared with fractures observed during the last 2 years before inclusion, vertebral fracture rates decreased (OR = 0.61 [0.47, 0.80], *P* < .001) across all years compared with baseline (Fig. 2A). Vertebral fractures exhibited consistent reductions ranging between 32% in years 1-2 to 72% in years 7-8 (Table 2). Similarly, nonvertebral fracture rates also decreased (OR = 0.23 [0.18, 0.31], *P* < .001) across all years compared with baseline, with the largest effect in the first 2 years, and then persisted at subsequent follow-ups (Fig. 2B). Nonvertebral fracture rates were lower at all follow-ups vs. baseline and showed consistent reductions ranging between 75% in years 1-2 and 68% in years 7-8 (Table 2). We observed no differences in the effects of ZOL treatment on fracture rates between the different follow-ups (Fig. 2A and B).

In sensitivity analysis, age, sex, and total hip BMD did not alter the effect of ZOL treatment on vertebral fracture rates. However, total hip BMD was a significant predictor of nonvertebral fracture rates, with 1 SD increase in total hip BMD being associated with lower rates of nonvertebral fractures (OR: 0.21[0.07, 0.61], *P* = .005).

## Discussion

This study demonstrated that a treatment strategy employing intermittent dosing of ZOL based on a 35-μg/L cut-off for the bone turnover marker S-P1NP provided consistent BMD increases, reduction in bone markers, and sustained fracture reduction. With this approach, only 39% of the patients needed more than one infusion during a mean follow-up time of 4.4 years. This approach could potentially result in reduced costs related to drug expenses as well as costs related to clinical follow-up.

The data emerging from this study demonstrate that a follow-up using BTMs every 1-2 years for the timing of a new ZOL infusion is sufficient to maintain treatment efficacy. As shown in Fig. 1, less than half of the patients needed a new infusion at the different follow-up points, using this strategy. Sustained BTM suppression and fracture risk reduction following one ZOL infusion were established from previous studies.^6-8^^,^^16^

P1NP was chosen as the primary response variable over CTX for the following reasons: (1) our studies showed the absence of significant diurnal variation for S-P1NP, be it in treatment naïve patients or even less so in patients on antiresorptive therapy, permitting sampling regardless of time of the day.^4^ On the contrary, CTX displays diurnal variation in treatment naïve patients and is stable only in patients on antiresorptive therapy. (2) We chose to use a cut-off of 35 μg/L for S-P1NP as previously proposed by Naylor et al. in the TRIO study.^15^ They showed that the patients who had the effect of bisphosphonate treatment by means of reduction of S-P1NP exceeding the range of least significant change, corresponded to the patients reaching BTM values in the lower half of the premenopausal reference interval. In a substudy form the Norwegian Capture the Fracture Initiative (NoFRACT), finalized after initiation of this study, it was demonstrated that BTMs in the lower half of the premenopausal reference range corresponded with a higher increase in BMD, and lower S-P1NP was associated with lower fracture rate.^4^

BMD exhibited a larger increase during the first 2 years than in years 4-8, with the largest increases in the spine, and remained stable during the observation time. This was also found in the post hoc analyses from the HORIZON-PFT trial, where patients who got one single dose of ZOL were compared with placebo.^6^ However, these patients were older (75.7 years) and had a lower BMD T-score at the hip of −2.7 and a high rate of vertebral fractures (66.5%). In these patients, BMD increased by 4% in the lumbar spine and 2% in the femoral neck. In our population, the BMD increase in the spine exceeded 6%, and the increase at the hip was 2.6%.

About half of the patients had sustained previous fractures, and the vertebral fracture rate was high in the 2 years before the first infusion. This was as expected since all the patients fulfilled the criteria for ZOL treatment but also imply a high imminent fracture risk in these patients must be taken into consideration when interpreting the actual decrease in fracture incidence during the observation period. Even so, the overall fracture rates decreased with 39% for vertebral fractures and 77% for nonvertebral fractures compared with the fracture rate the 2 years preceding baseline. Although not directly comparable, the clinical fractures HORIZON-PFT trial decreased by 32% and 34% over 3 years in the groups receiving a single infusion of ZOL vs. once yearly compared with placebo.^6^ The 2-year reduction of morphometric vertebral fractures (23%) was lower than reported in the HORIZON-PFT trial (71%),^17^ but vertebral fracture reductions exhibited a continuous further decrease to 72% in years 7-8. The primary reason for this discrepancy is probably incomplete sampling of vertebral fractures in the 2 years before treatment. This notion is further corroborated by the fact that further decreases were demonstrable as the trial proceeded, where regular VFA data were available. Compared with the HORIZON-PFT trial, our cohort exhibited a more pronounced reduction in nonvertebral fractures (25% vs. 75%, respectively). One possible explanation for this discrepancy is the very high baseline nonvertebral fracture rate in our cohort (46.5%) compared with a lower incidence in the HORIZON-PFT trail (placebo rate 10.5% over 3 years). The number of nonvertebral fractures increased slightly at the end of the study period, but it has to be taken into account that the study sample was small at years 5-8 and the patients were older than at baseline with a mean age of 80.3 years and therefore had a higher risk of fractures.

BMD in this study was not as low as in many patients who are offered ZOL worldwide. However, at baseline, mean BMD T-score at the lumbar spine was −2.4 and mean age was 68 years, and the fracture rate was high. We believe that this cohort is representative of typical osteoporosis clinics across the world. In the last decade, the focus on secondary fracture prevention has elucidated the need for treatment in patients despite a BMD T-score above −2.5, and Reid et al. ^18^ has shown the efficacy of ZOL also in women with osteopenia, with significant lowering of fracture rate with ZOL administration every 18 month compared with placebo. Many patients cannot have oral bisphosphonates due to intolerance, gastrointestinal issues, or poor compliance, and often an anti-osteoporosis drug with rapid effect is warranted due to high imminent fracture risk, for instance after a hip or vertebral fracture. On the other hand, the treatment strategy used in our study was not applied to patients with very low BMD T-scores or several recent vertebral fractures, as those patients were offered osteoanabolic treatment instead. The approach of this study is therefore not generalizable to patients with the most severe osteoporosis. Dosing of ZOL based on S-P1NP is mostly applicable in patients who do not have conditions that falsely influence BTMs such as severe renal impairment, or in patients using glucocorticoids. The safety of infrequent dosing in women with osteopenia has been demonstrated in the 10-year observational study of Reid et al. The women who had got ZOL every 18 month for 6 years had a reduced fracture rate up to 3.5 years after the last dose of ZOL, but not thereafter.^19^ They studied the correlation between BTMs and BMD loss in a subset of 50 patients and could not conclude that BTMs were suitable for making treatment decisions in individual patients, but the sample was small.

This study has several limitations. The lack of a control group is of importance. Due to the retrospective design and the clinical setting, we did not have a proper control group. We therefore used the method that has been most often adopted in real-life experience studies, where no control group is available, namely to compare fracture incidence over a period before treatment to fracture incidence after the institution of treatment.^20^^,^^21^ In addition, the study sample was small, especially the number of patients at the two last time points, and these results must be interpreted with caution. The retrospective design of the study is also a limitation. However, the data were collected from a real-life cohort with an original research question and a practical approach.

We therefore believe that this treatment approach has many benefits that ideally should be confirmed in larger prospective randomized trials, comparing ZOL given once yearly with ZOL given once followed by yearly measurement of S-P1NP for the timing of the next infusion. If our results are confirmed, this could potentially lead to a significant change in clinical practice with potentially less frequent administration of ZOL. The use of BTMs as a surrogate for treatment effect can also extend the intervals between DXA scans during follow-up.

## Conclusion

This study demonstrated that it is possible to achieve a reduction in bone markers, consistent BMD increases, and sustained fracture reduction, by dosing ZOL intermittently based on bone marker cut-offs. This approach could potentially result in reduced costs related to drug expenses, reduced costs related to clinical follow-up, and improved treatment adherence. Exposure to lower total doses of bisphosphonate might also potentially reduce the risk of rare side effects such as osteonecrosis of the jaw and atypical femoral fracture. However, further large-scale data, ideally with a control group, are required.

## Author contributions

Tove Tveitan Borgen (Conceptualization, Data curation, Formal analysis, Investigation, Methodology, Software, Visualization, Writing—original draft, Writing—review & editing), Sindre Lee-Ødegård (Formal analysis, Methodology, Software, Visualization, Writing—review & editing), Barbara Fink Eriksen (Data curation, Writing—review & editing), and Erik Fink Eriksen (Conceptualization, Data curation, Investigation, Methodology, Project administration, Resources, Writing—original draft, Writing—review & editing)

## Funding

None declared.

## Conflicts of interest

T.T.B. reports speaker fees from UCB, Amgen, Roche Diagnostics, and Pharma Prim. Advisory board for UCB. S.L.Ø. reports speaker fees from Novo Nordisk and Eli Lilly and is on the advisory board for Helseresepten. B.F.E.: none. E.F.E. reports consultant fees from UCB, Amgen, Gilead, and Pharma Medico, and honoraria from Amgen, Gedon Richter, and Novo Nordisk. Study support from Roche Diagnostics.

## Data availability

The data underlying this article cannot be shared publicly due to the privacy of individuals who participated in the study. The data will be shared on reasonable request to the corresponding author.
